# Fostering open science and responsible research practices: A pre-post study

**DOI:** 10.12688/f1000research.155832.1

**Published:** 2025-03-24

**Authors:** Jaisson Cenci, Marcos Britto Correa, Lex Bouter, David Moher, Ewald Bronkhorst, Marina Christ Franco, Fausto Medeiros Mendes, Tatiana Pereira-Cenci, Marie Charlotte Huysmans, Maximiliano Sérgio Cenci

**Affiliations:** 1 Dentistry, Radboudumc Department of Dentistry, Nijmegen, Gelderland, 6525EX, The Netherlands; 2 Dentistry, Universidade Federal de Pelotas, Pelotas, State of Rio Grande do Sul, 96015560, Brazil; 3 Philosophy, Vrije Universiteit Amsterdam, Amsterdam, North Holland, 1081 HV, The Netherlands; 4 Clinical Epidemiology, Ottawa Hospital Research Institute, Ottawa, Ontario, Canada; 5 Dentistry, Universidade de Sao Paulo, São Paulo, State of São Paulo, Brazil

**Keywords:** open science, research integrity, educational intervention

## Abstract

**Background:**

Educational initiatives could foster the adoption of open science (OS) and responsible research practices (RRPs). This single group pre-post study evaluated the impact of an educational intervention on increasing the adherence, knowledge and perceptions about adopting OS practices and RRPs among graduate researchers at a Brazilian University.

**Methods:**

Graduate students from a southern Brazilian university were invited to participate in a course addressing OS and RRPs. The intervention was an online interactive course on OS and RRPs. The number of OS outputs, including Open Science Framework (OSF) accounts, study registrations, protocols, analysis plans, data sets, preprints, and the number of projects published by each participant were collected before and after the intervention. Additionally, a self-administered online questionnaire was applied before and after the intervention to evaluate participants’ perceptions on RRPs, OS practices and on the current researchers’ evaluation system.

**Results:**

Eighty-four students finished the course and 80 agreed to participate in the study. The number of OSF accounts increased from 7 to 78 after the course, and the number of projects increased from 7 to 10, six months after the intervention. No registrations, protocols, analysis plans, data sets, or preprints were found after 6 and 12 months, respectively. The participants’ perceptions of the current research evaluation system and on the OS practices and RRPs changed positively with the intervention. Also, the intention to adopt practices like registration, protocol and preprint publications has noticeably increased after the course.

**Conclusions:**

The number of participants’ OSF outputs showed little or no improvement after the intervention. The most important impact difference could be identified in terms of the participants’ perceptions and intentions to adhere to such practices in the future.

## Contributorship

Conceptualization: JC, MCF, DM, MSC

Methodology: JC, MCF, TPC, MBC, EB, DM, LB, MCH, MSC

Data curation: JC, MBC, EB

Writing-Original draft preparation: JC, MSC

Writing-Reviewing and Editing: JC, MBC, LB, DM, EB, MCF, FMM, TPC, MCH, MSC.

## Introduction

Fostering transparency and responsible research practices (RRPs) is a continuous goal in science and in any educational program designed to train future researchers. In this context, special attention has been given to initiatives that aim to promote RRPs and research integrity (RI). Also, it has been acknowledged that open science (OS) practices can help to foster RRPs and promote transparency in research. ^1^ While one of the goals of promoting RRPs is to prevent research misconduct, including falsification, fabrication, and plagiarism, recent evidence has pointed out that questionable research practices (QRPs), such as p-hacking and spin, are also harmful and responsible for impairing research quality and reproducibility. QRPs are more subtle and identifying them is more difficult. It requires having access to all stages of the research cycle, including well-reported registrations, full protocols, complete raw datasets, and other research materials: open science. ^2, 3^

In recent years, as a response to the so-called reproducibility crisis, a growing interest in meta-research has been observed. ^2, 4^ Several meta-research studies point to the current problems in the published literature, and this was also evidenced by the “reducing waste in research” movement. From the academic stakeholders’ perspective, one of the proposed interventions is promoting RI courses and training to their researchers. This type of intervention would promote more reflection and induce higher-quality and relevance in science, focusing on transparency, integrity, and scientific rigor. ^5, 6^

The early career researchers (ECRs), who have an important role in knowledge production and dissemination, and who will also become the decision-makers and promotors of institutional changes, have crucial importance in the process of normalizing the adoption of OS practices. ^7^ However, some barriers are reported for the implementation of OS practices, ranging from lack of incentives and recognition in term of career progression to absence of infrastructure and training to incorporate these practices in the routine of research development. ^7, 8^

Since educational interventions are one way to overcome the lack of training, they could promote the adoption of OS practices among researchers and help promote RRPs. ^9– 11^ Therefore, this study aimed to assess the effect of an educational intervention on the adhesion of graduate students to OS practices. Secondary aims were changes in the perception before and after the intervention in terms of adopting OS practices and RRPs in the scope of typical research activities to promote career and science advancements, social impact, and personal satisfaction. Additionally, changes in perceptions about the actual research evaluation system were assessed. The study hypotheses were that a) the intervention increases the adherence to OS practices measured by counting the open materials shared by the participants; and b) the knowledge and perceptions about RRPs and OS practices are improved by the intervention.

## Methods

### Trial design

A single-arm pre-post study was designed following the CONSORT (Consolidated Standards of Reporting Trials) statement. ^12^ The study protocol was approved by the Research Ethics Committee of the Faculty of Dentistry, Universidade Federal de Pelotas, Brazil, ( https://wp.ufpel.edu.br/cepesef/) on March 03, 2022, under tracking number 54276221.2.0000.5318. This study also adheres to the Declaration of Helsinki statement. ^13^ The protocol, questionnaire, analysis plan, Informed Consent Form (ICF), and the complete anonymized dataset are openly available in the project folder on the OSF platform ( https://osf.io/nz7va/) without restrictions. No significant methodological changes occurred after the study began, and it was conducted according to the registered protocol.

### Participants

The study was carried out at the Universidade Federal de Pelotas (UFPel) in southern Brazil. The graduate programs at UFPel comprised at the time of the study of 1265 Master’s and 1374 PhD candidates. In Brazil, academic and professional masters are part of graduate studies, and professional habilitation is received after the bachelor studies. At UFPel, no course about RRPs and OS was available to all candidates before the implementation of the course reported here.

The inclusion criteria were MSc and PhD candidates, registered in the graduate programs at UFPel. The exclusion criteria were students not fully enrolled in the programs, i.e, participating in specific classes but not fully enrolled, trainees, visiting scholars and mobility students.

### Recruitment and timeline

**Figure 1.**
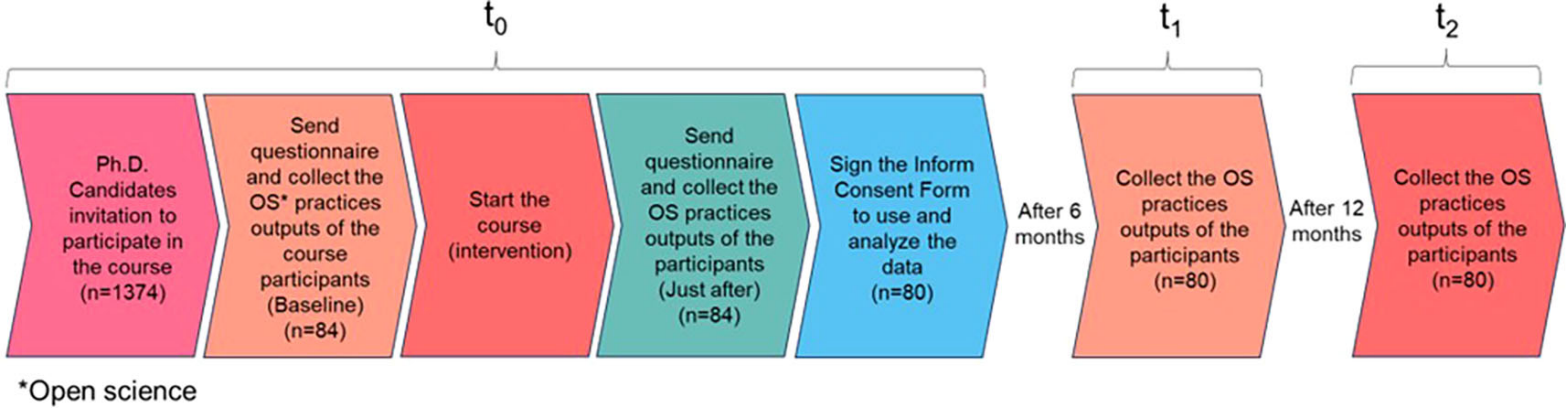
Participant timeline with the study steps described.

The inclusion criteria were applied to all MSc and PhD candidates at UFPel registered in the Brazilian national system and all of them were invited to participate. The list of all eligible participants and their e-mail addresses were obtained from the responsible department at UFPel (Pró-Reitoria de Pesquisa, Pós-Graduação e Inovação - PRPPGI). Before starting the course, the students who accepted the invitation were asked to answer a questionnaire (survey tool developed for this study and explained below) for the first time. In the same way, the number of OS practices outputs from each participant was collected at the Open Science Framework (OSF.io) before they started the course. Once all the enrolled participants answered the questionnaire, the course started. At the end of the course, all participants were invited to answer the questionnaire again ( Figure 1).

### Intervention

**Figure 2.**
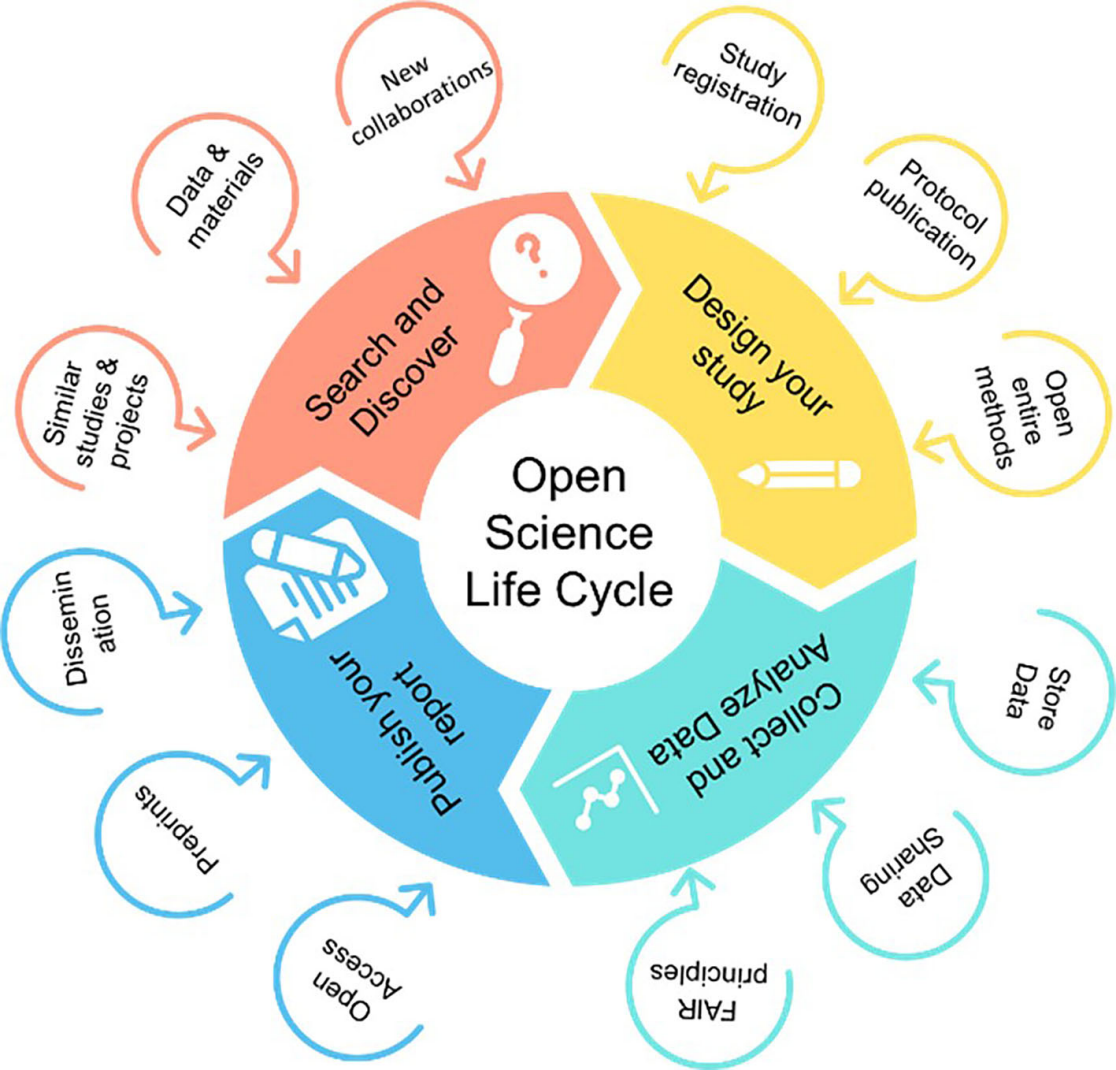
The steps and details of the OS life cycle (Adapted from https://www.cos.io/).

The intervention was an online interactive course focused on topics such as OS, including open data, RI, RRPs, and transparency in science. During the course, the main problems resulting from research misconduct and questionable practices were discussed, and possible solutions based on adoption of OS practices were promoted. The course also showed how the whole research lifecycle could be carried out using OS practices ( Figure 2).

The course was made available in video format with the participation of instructors and researchers on the topics, and included 85 hours of lectures, workshops, and supporting learning material (self-study). The box below provides a summary of the course structure and its contents.

Box 1. **Modules, main content, and activities of the course.**

**Table:** 

Module	Main content	Activities
1	Presentation of the general content, format and objectives of the course; explanation of tasks and assessments (asynchrony).	Answer a questionnaire
2	Introduction to RI, OS and RRPs; brief explanation about the OS life cycle; research misconduct and questionable research practices (asynchrony).	Using the support material provided, discuss the topics presented in class in the module discussion forum Answer 4 open questions about the subject
3	Reproducibility and replicability; reproducibility crises - definition, causes and possible interventions (asynchrony).	Writing a short text about which practices you should adopt to favor reproducibility Self-study with the support material Discussion forum
4	Study registration and protocol publication; open methods; what is Open Science Framework and how to use it	A synchronous workshop demonstrating all steps of the research life cycle, from registering and publishing protocols, to sharing the manuscript with preprint Students were invited to open an account on the Open Science Framework Self-study with the support material Discussion forum
5	Academic life and citizen science; Definition; Principles; types and examples of citizen science; benefits; challenges; citizen science in practice - the academic vision; citizen science in practice - the view from the other side (asynchrony).	Write a short text about academic life and citizen science, and what it is relation with OS and RI Self-study with the support material Discussion forum
6	Open data and data sharing; Why, where and how to share data; FAIR date; data repository (asynchrony).	Write a short text about the importance of data sharing, and what it is relation with OS and RI Self-study with the support material Discussion forum
7	Researcher assessment; what the current evaluation of researchers is like; influence of current rewards on the integrity of science; main initiatives; institutions already embracing the change (asynchrony).	Write a short text about the influence of the researcher evaluation format on OS and RI adoption Self-study with the support material Discussion forum
8	Synchronous workshop to answer questions	Active participation and answering a questionnaire

Students were able to access the videos through login into the platform. The intervention was applied just once to each participant, and at the end of the course, each participant was invited to answer the questionnaire again.

The course syllabus, all course materials (in Brazilian Portuguese), and content are openly available on a specific project hosted at the Open Science Framework ( https://osf.io/7rz5n/).

### Data collection

OSF data collection

According to the primary objective of our study, data related to the participants’ OS practices outputs was collected at the OSF website (osf.io). This platform was chosen because the participants had no prior knowledge of any OS tools, and the OSF was demonstrated during the course. The procedure for data collection was based on typing the participants’ names directly on the OSF searching tool. If the participant had an OSF account, this was registered, as well as the number of registered studies, protocols published, analysis plans related to their projects, data sets shared, preprint publications, and OSF projects for each participant.

Questionnaire

Following an extensive literature review conducted by JC ( https://osf.io/hjtwn), only one relevant but unvalidated questionnaire was identified. ^14^ This questionnaire was then adapted to the Brazilian context, incorporating elements from the Brazilian research assessment system and Open Science (OS) practices based on indicators proposed in the Declaration on Research Assessment (DORA), ^15^ the Leiden Manifesto, ^16^ and the Hong Kong Principles. ^17^ The final survey comprised 50 questions distributed across five sections.

The entire questionnaire, including the introduction, informed consent form, questions, and explanation of the concepts, is available in the project folder on the OSF platform ( https://osf.io/nz7va/). In brief, the questionnaire consisted of: Part 1 - demographic questions; Part 2-3 questions on researchers evaluation in Brazil; Part 3-27 typical academic activities or characteristics, for which respondents were asked to rate the perceived impact on (I) advancing career, (II) advancing science, (III) personal satisfaction, and (IV) social impact; Part 4-4 questions about their perception about the freedom they sense to adopt RRPs and OS practices, and their willingness to adopt those practices it in the future; and Part 5 - questions related to their current scientific production. Only the 3 first parts of the questionnaire are reported here, because they answer the stated secondary objectives of our protocol.

The questionnaire was hosted online at REDCap (REDCap – Research Electronic Data Capture, Vanderbilt, Nashville, Tennessee, USA, www.projectredcap.org/). REDCap was used to collect, store, and anonymize the data.

Piloting the questionnaire

This questionnaire was tested with 93 different master’s and PhD students from UFPel (who did not participate in the final study) to assess its comprehensibility in generating answers about the research hypotheses. For each question, participants were asked to answer whether the question was clear about their understanding when answering it. The questions evaluated with a low level of clarity and understanding were edited until they reached a consensus among the participating researchers. The total time needed was about 20 minutes. The pilot results are available at OSF ( https://osf.io/kag4w).

### Outcomes

Primary outcome

The primary outcome was the number of outputs per participant on the OSF platform (preregistrations, protocols, analysis plans/data management plans (DMP), data sets and preprints), before and after the intervention. The primary outcome was collected by JC before the course, just after, and after 6 and 12 months.

Secondary outcomes

1 - The percentage of graduate candidates at UFPel satisfied and dissatisfied with the current evaluation processes for researchers in Brazil before and after the intervention, assessed through the participant’s answers to the 3 questions of the second part of the questionnaire.
2 - The changes in the participants’ perceptions on the importance of typical research activities on four domains (advancing career, advancing science, personal satisfaction, and social impact) before and after the intervention, assessed through the participants’ answers to part 3 of the questionnaire;

### Blinding and consent

The participants were unaware of being enrolled in our study during the course. At the end of the intervention, and after they had answered the questionnaire for the second time, they were informed about the intention of reporting on the data collected, and they were invited to participate and sign the informed consent form (ICF). We used only data from participants who gave permission. There were no negative consequences for students who refused, as the students’ evaluations and grades were conducted and finished before we asked for their ICF signatures.

### Statistical methods

Descriptive statistics was used to report the number of OS practices and summarize questionnaire answer’s part 1. In order to confirm the results for the primary outcome, the Friedman test with Wilcoxon signed-rank with Bonferroni correction post hoc was applied

To answer the secondary objectives, the questionnaire answers were evaluated as follows:

Descriptive analysis was performed to get the frequencies of the participant’s answers to the 3 questions addressing their perceptions of the current researchers’ assessment system (Charts 2, 3, and 4). No statistical test was previous planned in the protocol, but to confirm the differences in the results, the Wilcoxon signed rank test with continuity correction was applied for each question in part 2. To make evaluation possible using the Wilcoxon test, considering alternatives A, B, C and D as an ordinal scale, alternative E was treated as missing for question 3 of part 2.

To compare the participants’ perceptions about the 27 common researchers’ activities related to the third part of the questionnaire, those activities were separated into activities related to traditional and non-traditional practices, as indicated in Table 1. The main objective was to identify what activities resulted in essential, important, irrelevant, unfavorable, or detrimental perceived impact in each one of the 4 domains (advancing career, advancing science, personal satisfaction, and social impact), and what changes related to it we can identify comparing before and after the intervention. To enable statistical analysis, the responses related to the Likert scale were transformed numerically as follows: detrimental = 1, unfavorable = 2, irrelevant = 3, important = 4, essential = 5. A score (average) for each group of activities was calculated and compared ( Table 2). The nomenclature and characterization of activities into traditional and non-traditional were adapted from previous studies. ^18, 19^

**Table 1.** Traditional and non-traditional activities according to the questionnaire.

Non-traditional	Traditional
Publishing in open-access journals; Expertise in peer review/editing; Replication of studies; Publish findings that didn't work; Sharing the complete data and detailed methods; Reviewing raw data from students and staff is; Conducting innovative research with a high risk of failure; Collaborating across borders, disciplines, and sectors; Having your papers read and downloaded Having a public reach, in social media, news, etc.; Having your results and findings used or implemented in practice; Activities in knowledge synthesis such as systematic reviews and meta-research; Development and publication of study protocols; Sharing data in open-access repositories is; Knowledge translation activities; Publish the project analysis plan openly is; Acting on extension projects; Publishing preprints; Participating and presenting results at scientific meetings; Direct student supervision.	The publication of scientific papers; Posting comments or editorials; A large number of published papers; Publication in high-impact factor journals; Publishing more scientific papers than other researchers; Connecting to renowned researchers; Being cited in the scientific literature;

**Table 2.** Scores and the confidence interval for each domain on Traditional and Non-Traditional groups.

	Traditional				Non-Traditional			
	Averages		Paired T-Test		Averages		Paired T-Test	
	Before	After	Average difference (CI)	p	Before	After	Average difference (CI)	p
** career advancement**	4,18	4,24	-0.6 (-0.15, 0.03)	0.20	4,26	4,32	-0.06 (-0.13, 0.01)	0.11
**advancing science**	4,13	3,96	0.18* (0.09, 0.27)	<.01	4,51	4,61	-0.10* (-0.15, -0.03)	<.01
**personal satisfaction**	4,03	4,01	0.02 (-0.07, 0.10)	0.67	4,22	4,30	-0.07 (-0.14, 0.003)	0.06
**social impact**	3,93	3,81	0.13* (0.03, 0.22)	<.01	4,37	4,54	-0.17* (-0.23, -0.11)	<.01

Values in “before” and “after” are averages. Abbreviations: CI, confidence interval; p, p value; *, statistical significance (p value < 0.05).

Although no statistical test was planned to this part in the protocol, a Paired T-test was performed for each domain (advancing career, advancing science, personal satisfaction, and social impact) in each group (traditional and non-traditional) to aid data interpretation.

Despite not being listed in the protocol as one of the objectives of this study, the assessment of the intention to adopt OS practices (protocol registration; protocol publication; opening the analysis plans; opening the research notebooks; data sharing; and sharing codes), assessed by a question in part 4 of the questionnaire, presented interesting results and was therefore included in this article. Descriptive analysis was done to analyze the answers regarding the participants’ intention to adhere to these practices in the future, and charts was presented to summarize the results ( Figure 7). The Wilcoxon test was also applied to each practice to confirm the results.

A confidence interval of 95% and a significance level of α = 0.05 were used for all quantitative analyses. All analyses were conducted using R statistical software (Version 4.3.0, RStudio Inc., Boston, USA, free available at https://www.r-project.org/).

## Results

The course took place from October 15th to December 14th, 2022. The questionnaire was applied before the course, from October 5 ^th^ to 14 ^th^, and reapplied after the course, between December 14 ^th^ to 27, 2022.

The data relative to their OSF outputs were collected on October 4th and December 14th, 2022, for the before and just after evaluation; on July 15th, 2023, for the 6-month follow-up; and on January 22nd, 2024, for the 12-month follow-up.

### Demographics

Of the 84 participants enrolled in the course, 83 finished all activities. Only the 80 participants who signed the ICF were included in the analysis. The mean age of the participants was 32.3, varying from 22 to 57 years. The gender distribution was 62 females (77.5%) and 18 males (22.5%).

Most participants were from STEM (Science, Technology, Engineering, and Math) graduate programs (62; 77.5%) and 18 (22.5%) were from the Arts and Humanity graduate programs. 37 (46.3%) participants were at the PhD level and 43 (53.8%) were at the MSc level. Most of the participants graduated from public universities (59; 73.8%), followed by private universities (20; 25.0%) and Community universities (1; 1.3%). About the duration since they acquired their last title (undergraduate or master’s graduation), 66 (82.5%) got it less than 5 years ago, 11 (13.8%) between 5 to 10 years, and 3 (3.8%) got it more than 10 years ago. Finally, most participants (73; 91%) had never taken a course addressing RI and OS practices.

### OSF outputs

**Figure 3.**
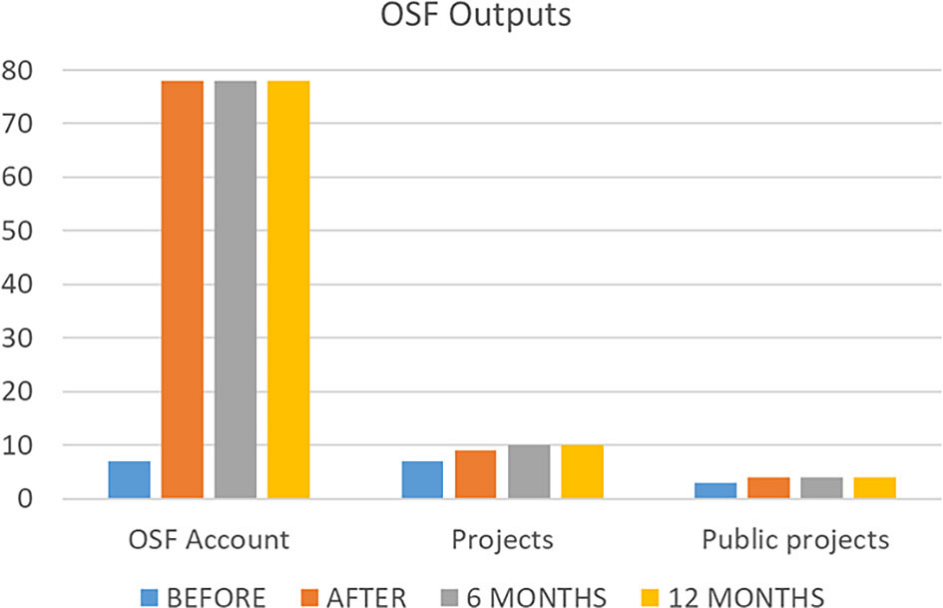
The number of OSF outputs before, just after, and six months after intervention.

To answer the study’s primary objective, the data referring to the number of outputs in the OSF platform was shown in Figure 3. The number of OSF accounts increased from 7 to 78 after the end of the course. Two participants have not made an account during the course, and this situation remained after 6 and 12 months. Before the course, the number of projects was 7 and the number of public projects was 3. Just after the course, they increased to 9 and 4 respectively, and after 6 months the number of projects increased to 10. No changes happened after 12 months of the end of intervention. No registrations, protocols, analysis plans, data sets, or preprints were found before or after the course.

The Friedman test with Wilcoxon signed-rank post hoc results shown that OSF account before the course is statistical different of the other 3 times evaluated (p<.01); the projects and public projects did not show any statistical difference between the evaluated time frames (p=0.6; p=0.3).

### Participants’ perceptions of the current researchers’ assessment system

**Figure 4.**
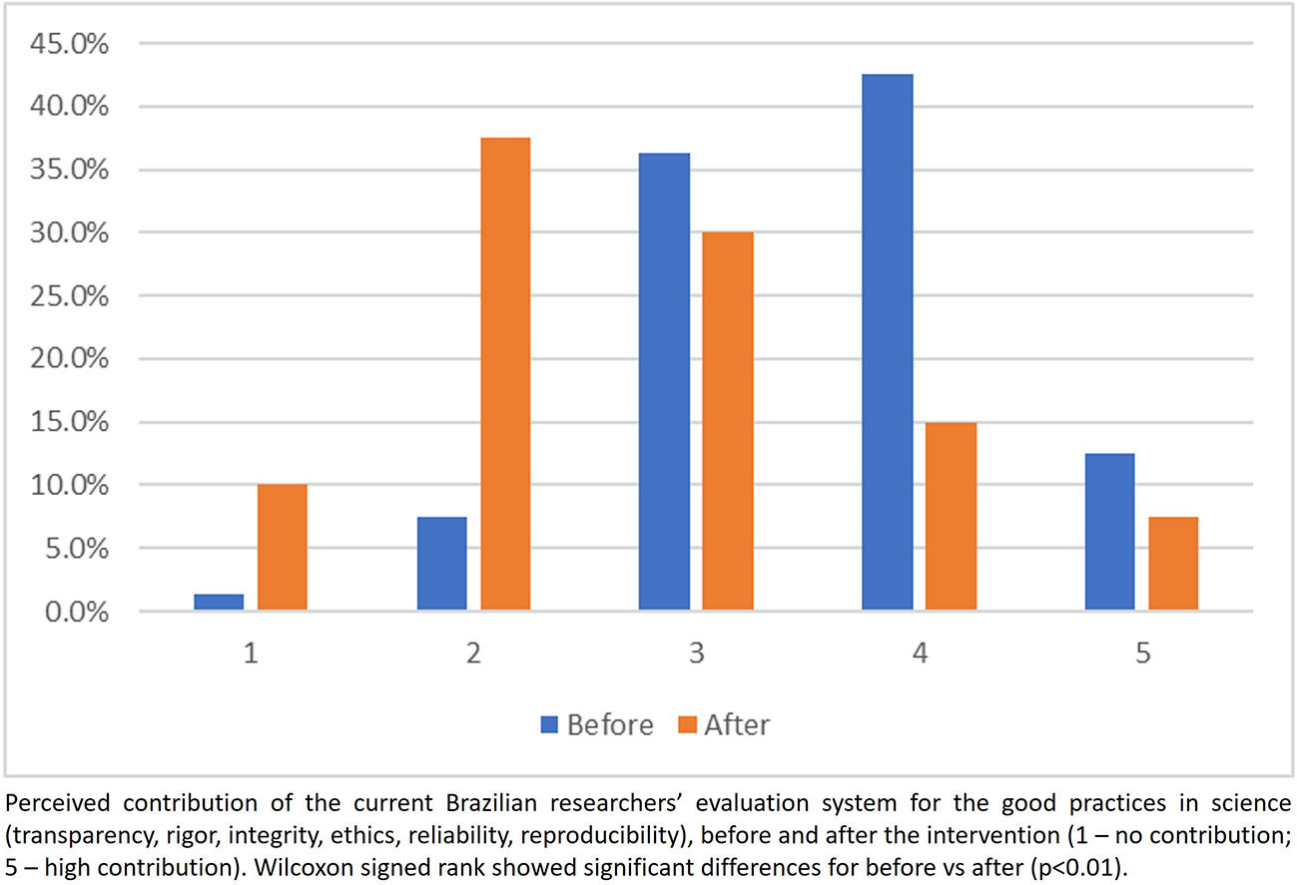
Participants answer to the question 1, Part 2 of the questionnaire.

**Figure 5.**
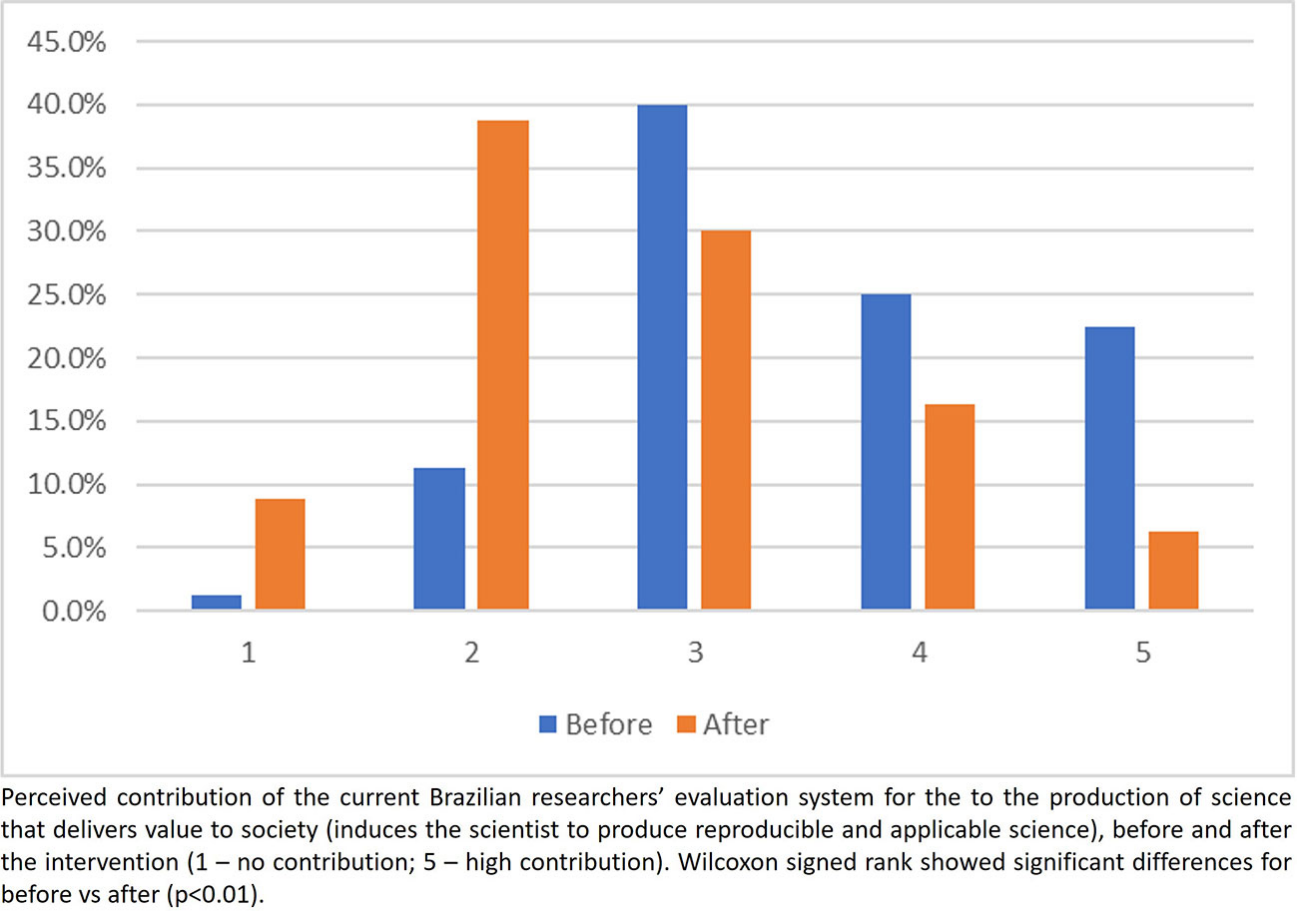
Participants answer to the question 2, Part 2 of the questionnaire.

Overall, participants perceived before the intervention that the current evaluation system contributes to promote good practices in research ( Figure 4). However, this perception changed after the intervention, showing a higher proportion of scores 1 and 2 (assessment system does not contribute) and a lower proportion in scores 4 and 5 (assessment system contributes to good practices in research) ( Figure 4). The same trends were observed in answering the question on how much the current evaluation system contributes to the delivering value to society ( Figure 5).

**Figure 6.**
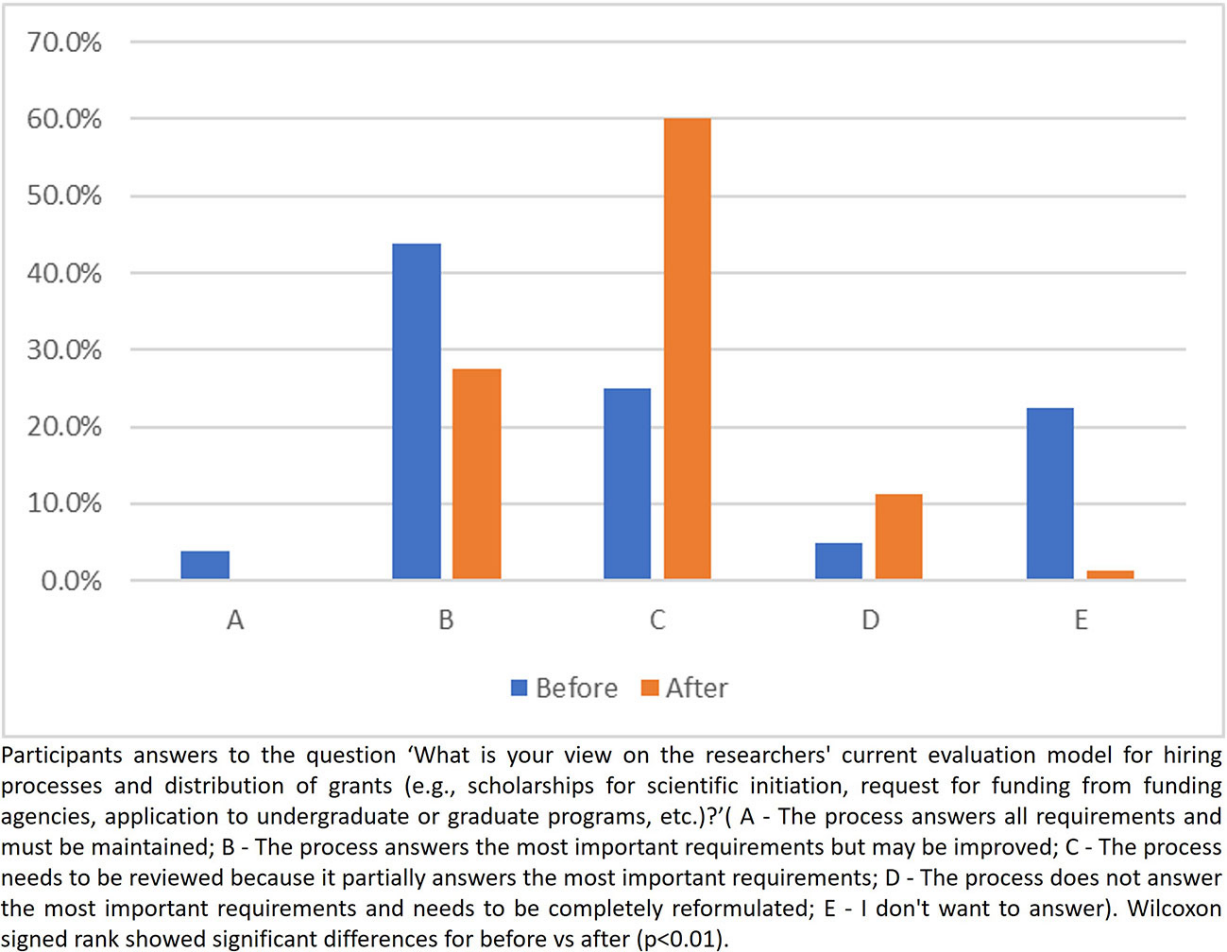
Participants answer to the question 3, Part 2 of the questionnaire.

When inquired about the relation between the researchers’ current evaluation model and the hiring processes/distribution of grants, no participant pointed out that ‘the process answers all requirements and must be maintained’ after the intervention compared to 3.2% in the first evaluation. The number of participants who answered that ‘The process answers the most important requirements but may be improved’ decreased from 43.8% to 27.5% after the intervention. The frequencies for the answer ‘The process needs to be reviewed because it partially answers the most important requirements’ increased from 25% to 60% and the answer ‘The process does not answer the most important requirements and needs to be completely reformulated’ increased from 5% to 11% after the intervention. The number of participants who chose the option ‘I don’t want to answer’ decreased from 22.5% to 1.3% ( Figure 6).

### Perceptions about the 27 common researchers’ activities

For both, traditional and non-traditional activities, there was a statistical difference in the importance given by the participants before and after the intervention only for ‘advancing science’ and ‘social impact’ domains ( Table 2).

### Current adoption or intention to adopt OS practices by the participants

**Figure 7.**
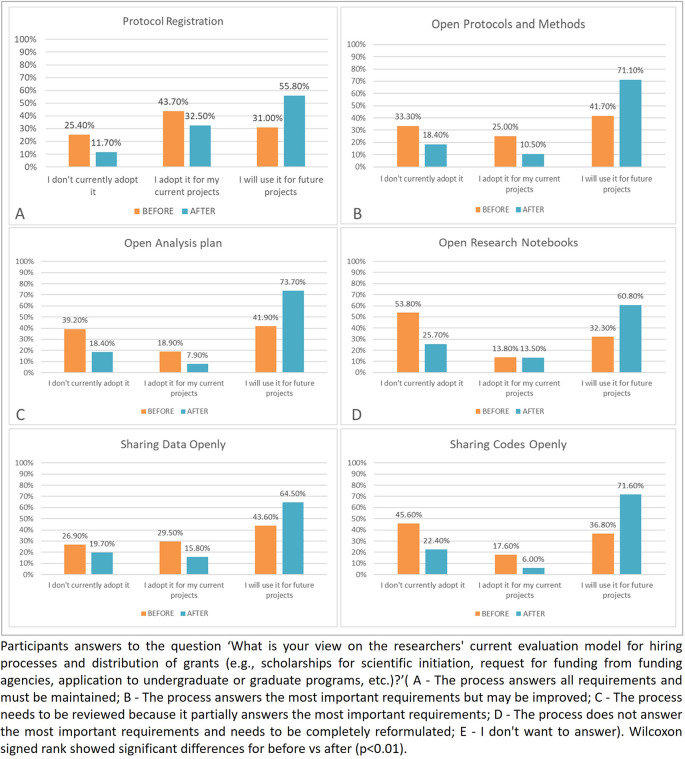
Participants’ intention to adhere to the open science practices in the future.

The answers to the question ‘What practices do you adopt or think about adopting for your projects?’ are shown in the Figure 7.

For the results of the Wilcoxon test, protocol registration (p<0.01), protocol publication (p<0.01), publishing and making available the analysis plans (p<0.01), opening the research notebooks (p<0.01), and sharing codes (p<0.01) showed statistical differences between before and after. Only the differences in data sharing (p=0.10) were not statistically significant.

## Discussion

To our knowledge, this is the first study exploring the impact of an educational intervention on the behavior and perceptions of graduate students in Brazil and South America. In that region, there is still less incentives to promote OS practices and RRPs in comparison with Europe and North America. ^20^ Despite education-based interventions being cited as an important driver for promoting RRPs and OS practices, ^11, 14^ the intervention applied to the participants in the current study proved insufficient for changing short-term behavior, showing little change after 6 and 12 months of follow-up. However, the intervention was somewhat effective to change perceptions of the impact of traditional and non-traditional research activities on advancing career, advancing science, social contribution, and personal satisfaction. Overall participants perceived that the non-traditional activities related to OS practices and RRPs are important to advance science and promote social impact. Concerning the perception about traditional academic activities, the intervention did not promote significative changes. The most significant impacts of the intervention were evident in how the participants perceived the current researcher evaluation system and their future intention to adhere to OS practices.

Adherence to OS practices increase after the course only for the registrations at OSF. However, creating an OSF account was one of the tasks to be carried out by students in the course, demonstrating that the best incentive to adopt new practices is to make them normative. This supports the idea that the role in changing policies of institutions and funders are essential for inducing a broad adherence to OS practices and RRPs, which was previously showed. ^14, 17^ Regarding the lack of adherence to OS practices, our results are in accordance with previous studies. ^21^ The possible reasons could be, that the participants on this study were graduate students in a context where not so many scientific products as publications, projects, and datasets are usually produced in a 12-month period. Typically, in Brazil, graduate students spend their first months or years following courses, and dedicate time to their research in the last period of their trajectory. Other reasons could be that our intervention target a multidisciplinary audience, with participants from a diversity of fields of knowledge, and some of them were used to produce only a traditional monography as the product of their graduation trajectory.

Observing the responses after the intervention to the first two questions about the current system for evaluating researchers, participants seem to have better understood the restrictions of the current system in contributing to the adoption of good research practices and in inducing researchers to produce science that adds real value to society. Regarding the third question of this part, after the intervention, no participant checked the alternative which said that the process did not need any change. In addition, there was a decrease in the number of participants who believe that the process answered the most important questions but could be improved, as well as an increase in the number of participants who believe that the process is flawed and needs to be revised or even completely reformed. Another important change following the intervention was the number of participants who chose ‘I don’t want to answer’, which showed a significant drop after the intervention, indicating that the intervention was partially effective in changing perceptions and knowledge. This finding suggests that educational interventions should be implemented as a routine.

Behavioral changes are deemed to be difficult to realize because they face several barriers and demand strong facilitators. There are many reasons why those changes take extra time and effort: usually the workflow of researches is already overloaded with activities, and important actions such as registering protocols or organizing datasets to be sharable take time, demand extra infrastructure, and most of the times there is no external incentive to these and other OS practices. Besides that, other studies have shown that educational training in ethics were appreciated by participants, ^21, 22^ but might not make researchers acting more ethical. However, courses combined with the proper incentives and means may play a crucial role in improving how research is done and the research culture, strengthening integrity rules, as well as preventing researchers from committing research misconduct. ^9^

When we assessed perceptions regarding 27 typical research studies, the perceived importance of traditional research decreased after the intervention, both for the advancement of science and for social impact, while the perceived importance of non-traditional research increased after the intervention. These results indicate an understanding that many traditional research activities do not exactly contribute to the advancement of science and social impact what corroborate with other studies which questioned researchers at more advanced stages of their career about their perceptions regarding traditional and non-traditional practices. ^14, 23^ However, the lack of difference in perceptions about career advancement is somewhat worrying, since it is clear that most non-traditional practices, such as study registration and data sharing, do not promote career progression and are usually not taken into account in the hiring and input distribution processes. ^17, 24^ This reinforces the previous idea; thus, if an individual understands that the current system does not contribute to knowledge and social impact, even if it takes time, he/she could be more prone to change behavior and enhance OS practices.

Our results show that the intervention was promising in terms of the increasing participant’s willingness to change. The drop in the number of respondents who said they did not currently adopt the practices and those who said they already adopted such practices, followed by the increase in the number of participants stating that they intend to adopt the practices in the future, might be interpreted as readiness for change. Likewise, most participants did not even know exactly what the mentioned practices meant before the intervention. The only exception was data sharing, which showed no statistically significant differences after the intervention. This could possibly be explained as a more intricate practice that depends on infrastructure and should follow several rules from the research environment in which the researcher is inserted.

Our research has several limitations. The study was conducted at a single institution with different courses that have very different research approaches, including the way they produce and disseminate scientific findings. There was no control group for comparison. To address our primary objective, we used only one of several platforms for registering studies, protocols, and preprints. Although this platform was demonstrated and used in the course, other platforms might be better suited for different areas of knowledge. Course students might answer the questionnaire a second time based on what was taught in the course, not necessarily because they understood and intend to follow the principles, but to achieve a sufficient evaluation to pass the course. Additionally, it is expected that participants who applied for the voluntary course already have some affinity or interest in the subject. The instrument, although piloted, was not validated and requires further adjustments before being used again in a new study. Another limitation is the intervention itself, which needs improvement for future studies to be more effectively applied and tailored to specific audiences and related areas. ^4^ However, even if short-term results are not ideal, an intervention addressing the subject can be beneficial by publicizing and encouraging participants to discuss the topic among themselves, with their advisors, institutions, financiers, and other stakeholders.

In addition, the limited results can be explained by the fact that training alone is not enough to change behavior regarding RRPs and OS practices. ^11, 14^ It is also worth remembering that researchers’ behaviors are not only influenced by the course content but also by the research system and the local research culture, including the examples supervisors and colleagues give. ^11, 25, 26^ Therefore, effective change in behavior towards adoption of RRPs and OS practices is not an individual journey, and should involve cultural changes in institutions, funders and evaluation systems. ^2, 9, 26, 27^

## Consent statement

Participants’ consent was obtained with an online form applied after they answered the questionnaire for the second time. To be included in the study and in the final results, participants had to sign the Informed Consent Form (ICF) which is available at ttps:// doi.org/10.17605/OSF.IO/NZ7VA. This ICF was previously approved by the same ethical committee that approved the study protocol.

## Ethics and consent

A single-arm pre-post study was designed following the CONSORT (Consolidated Standards of Reporting Trials) statement ^12^ The study protocol was approved by the local Brazilian ethics committee (Research Ethics Committee of the Faculty of Dentistry, Universidade Federal de Pelotas, Brazil), on March 03, 2022, under tracking number 54276221.2.0000.5318. This study also adheres to the Declaration of Helsinki statement. ^13^ The protocol, questionnaire, analysis plan, written Informed Consent Form (ICF), and the complete anonymized dataset are openly available in the project folder on the OSF platform ( https://osf.io/nz7va/) without restrictions. No significant methodological changes occurred after the study began, and it was conducted according to the registered protocol. Informed consent was mandatory as the study involved participant data. It was obtained to ensure ethical compliance. Participants were informed and given the choice to consent, ensuring voluntary participation and ethical compliance. The written consent was obtained online through signing the ICF by the participant.

## Data Availability

Open Science Framework (OSF): Research integrity practices: A Pre-Post Study with Brazilian Graduate Students. https://doi.org/10.17605/OSF.IO/NZ7VA. ^28^ The “Data set” folder in the project contains the following underlying data:
− OSF_outputs.xlsx - Anonymized data for the participants products on Open Science Framework platform. − Part1.xlsx – Anonymized data for the participants answers to the questions of the questionnaire part 1. − Part2.xlsx – Anonymized data for the participants answers to the questions of the questionnaire part 2. −
Part3_N_trad.xlsx – Anonymized data for the participants answers to the questions of the questionnaire part 3, regarding to the non-traditional activities. − Part3_Trad.xlsx – Anonymized data for the participants answers to the questions of the questionnaire part 3, regarding to the traditional activities. − Part4_Q123.xlsx – Anonymized data for the participants answers to the questions 1, 2 and 3 of the questionnaire part 4. − Part4_Q4.xlsx – Anonymized data for the participants answers to the question 4 of the questionnaire part 4. − Part5.xlsx – Anonymized data for the participants answers to the questions of the questionnaire part 5. Data are available under the terms of the Creative Commons Attribution 4.0 International license (CC-BY 4.0). Study materials are also included in the same project as follows:
− “Pilot” folder contain the pilot results of the questionnaire. − “Protocol” folder contain the study protocol, Informed consent form (ICF), and the questionnaire. − “Course Program and Details” folder contain the syllabus and other details about the educational intervention applied to participants.
