# Supplementary material for: Fostering open science and responsible research practices: A pre-post study
Source: F1000Res. 2025 Oct 20;14:318. Originally published 2025 Mar 24. [Version 2] doi: 10.12688/f1000research.155832.2 (PMC12120411; doi:10.12688/f1000research.155832.2)
Supplement: Peer review history

# Reviewer report: Reviewer response for version 2

**DOI:** 10.5256/f1000research.189858.r425512

**Published:** 2025-10-31

**Authors:** Joachim Schöpfel

**Affiliations:** 1 University of Lille, Villeneuve-d’Ascq, France

The revised version addresses my previous comments and suggestions and appears suitable for indexing. Thank you.

Is the work clearly and accurately presented and does it cite the current literature?

Partly

If applicable, is the statistical analysis and its interpretation appropriate?

Yes

Are all the source data underlying the results available to ensure full reproducibility?

Yes

Is the study design appropriate and is the work technically sound?

Partly

Are the conclusions drawn adequately supported by the results?

Yes

Are sufficient details of methods and analysis provided to allow replication by others?

Yes

Reviewer Expertise:

information sciences

I confirm that I have read this submission and believe that I have an appropriate level of expertise to confirm that it is of an acceptable scientific standard.

# Reviewer report: Reviewer response for version 1

**DOI:** 10.5256/f1000research.171052.r385379

**Published:** 2025-06-02

**Authors:** Priya Silverstein

**Affiliations:** 1 Ashland University, Ashland, USA

This article examines a pre-post study evaluating an educational intervention’s impact on graduate students’ understanding of open science and responsible research practices. While the intervention had limited effect on actual implementation (within the period studied), it did lead to an increase in intention to adopt open science practices.

 I think the authors do a good job of identifying the limitations of the study. I also agree with Sarahanne that the Registered Report format would have been preferable to be able to resolve some of the larger design concerns before data collection. 

 Nevertheless, I will focus on things the authors can change within the write-up itself, now that the study has been completed.

 1. I think the description of the increase in OSF accounts is misleading -- we later find out that students has to make an OSF account as part of the course itself, so I don't actually think this makes sense as an outcome measure. Instead, perhaps it would make sense to frame it as "despite the vast majority of students instructed to make an OSF account during the course doing so, [results about outputs on OSF]". So, the course helped them with one barrier to sharing outputs on the OSF (creating an account), but this wasn't enough to push them to share outputs.

 2. There is currently little rationale for the time points chosen for follow-up. It's ok if the rationale is logistical, but then this should be made clear.

 3. It would be helpful to have some detail about how the course was developed and by whom.

 4. The course is a great undertaking! The authors may consider sharing the course with FORRT (https://forrt.org/) so that it can be disseminated widely and further refined in future versions.

 5. The sentence "This platform was chosen because the participants had no prior knowledge of any OS tools" on page 6 is misleading -- there's no way of knowing that participants had no prior knowledge of OS tools, and there were a few accounts already, so at least some did have knowledge of the OSF. One rationale for the OSF is that it is a "one stop shop" for many different practices, rather than needing to learn multiple tools in order to engage in open science.

 6. The distinction between traditional and non-traditional seems a bit forced and the rationale for this split and definitions / conceptualisations could be clearer.

 7. There isn't a rationale provided for why some of the scales go from "does not contribute" to "contributes to good practices" -- isn't it possible that participants think these things contribute to BAD practices?

 8. Figure 7 caption is wrong -- same as Figure 5

 9. "However, creating an OSF account was one of the tasks to be carried out by students in the course, demonstrating that the best incentive to adopt new practices is to make them normative. This supports the idea that the role in changing policies of institutions and funders are essential for inducing a broad adherence to OS practices and RRPs, which was previously showed." -- I'm not sure about this, doesn't this just show that if you ask students to do something as part of the course they'll do it?

 10. "This finding suggests that educational interventions should be implemented as a routine." -- I'm not sure this conclusion follows.

 11. It would be good to introduce something about models of behaviour change (e.g. the COM-B model) when talking about behavioural change in the discussion. Something that might be interesting (although take with a pinch of salt as it is my own paper) is Silverstein et al. (2024) where we show that capability, opportunity, and motivation to engage in open research practices all affect engagement in open research practices. We found automatic motivation to be the strongest -- that is, having open research as part of your research routine and habits.

Is the work clearly and accurately presented and does it cite the current literature?

Partly

If applicable, is the statistical analysis and its interpretation appropriate?

Yes

Are all the source data underlying the results available to ensure full reproducibility?

Yes

Is the study design appropriate and is the work technically sound?

Partly

Are the conclusions drawn adequately supported by the results?

Partly

Are sufficient details of methods and analysis provided to allow replication by others?

Yes

Reviewer Expertise:

metascience, open science

I confirm that I have read this submission and believe that I have an appropriate level of expertise to confirm that it is of an acceptable scientific standard, however I have significant reservations, as outlined above.

# Response

**Published:** 2025-10-16

**Authors:** Jaisson Cenci

**Affiliations:** Dentistry, Radboudumc Department of Dentistry, Nijmegen, Gelderland, The Netherlands

**Dear** **Dr. Priya Silverstein,**

 Thank you very much for your thoughtful and detailed comments. Your feedback has been very helpful in improving the clarity and rigor of our manuscript. Below, we outline the changes made in response:

- We agree with your point that the increase in OSF accounts should not be interpreted as an outcome. We have included an extra explanation to clarify that creating an OSF account was a course requirement and used this to reflect on remaining barriers to the actual implementation of OS practices in the limitation section.
- Regarding the rationale for the timepoints, we chose these follow-up timepoints, acknowledging the logistical and institutional constraints involved.
- About the course dissemination and development, we have included details about the development team and the conceptual basis of the course, as well as made the course  openly available on OSF
- We reworded the ‘data collection’ section to reflect that while most participants were unfamiliar with OS platforms, a few did have prior experience. The rationale for choosing OSF is now more straightforward.
- Traditional vs non-traditional activities were elaborated on the rationale of this distinction and its foundation in prior literature and initiatives advocating for more responsible research practices.
- We clarified the intent of the 1–5 scale regarding participants’ perceptions of whether the system contributes to *good* practices, and not whether it promotes *bad* ones.
- The figure captions have been corrected. Thank you for spotting this.
- We integrated a brief sentence in the discussion to contextualize the observed gap between intention and behaviour. We found the insights of your 2024 article valuable. Thanks for mentioning it.

Once again, thank you for your detailed and constructive review. We believe these changes have significantly improved the quality and clarity of the paper.

 Kind regards,

 Jaisson Cenci (on behalf of all co-authors)

# Reviewer report: Reviewer response for version 1

**DOI:** 10.5256/f1000research.171052.r375971

**Published:** 2025-05-28

**Authors:** Sarahanne M Field

**Affiliations:** 1 University of Groningen, Groningen, The Netherlands

This article presents a timely and relevant investigation into the impact of educational interventions on graduate students' awareness and adoption of open science and responsible research practices. In my perspective, it is a valuable contribution to research on open science education, particularly within a Global South context. It is well-conceived and well-reported but would benefit from methodological enhancements and more robust behavioral measures in future work. The strongest impact observed is in the shifting of attitudes, suggesting that this course could serve as a solid first step in a larger institutional transformation process, provided follow-up mechanisms and structural incentives are also put in place.

 While the topic and approach are highly relevant, particularly in regions where OS adoption is still emerging (currently still a huge gap in the literature, and a generally understudied issue), the study's design limits its ability to draw strong causal conclusions. This is clearly pointed out in the article, and I really appreciate the authors not trying to oversell their conclusions.

 This said, the absence of a control group makes it difficult to attribute the attitudinal changes that were observed solely to the intervention. Introducing a comparison group in future research would allow for more definitive evaluations of effectiveness and rule out external influences as alternative explanations for the outcomes observed. Naturally, however, this is not something the current article can be changed to include (this would have been good as a registered report, because these kinds of things can be caught before data collection is made). 

 Although the intervention significantly improved participants' awareness and perceptions regarding OS and RRPs, it had a limited impact on actual behavior. Despite a substantial increase in OSF account creation (from 7 to 78, if I read it correctly) there was little follow-through in terms of registrations, protocols, preprints, or data sharing. This disconnect highlights a need for interventions that go beyond awareness-raising. Integrating structural incentives, such as recognition through academic credits, certificates, or institutional rewards, could promote meaningful engagement and sustained behavioral change. Furthermore, extending the follow-up period beyond 12 months may better capture the long-term influence of such training on research output, particularly as graduate students often take time before producing sharable research materials. I think these things can be better emphasised in the discussion. 

 The study’s sole reliance on OSF to assess behavioral change may underestimate the true extent of participants’ engagement with OS too, and this is something else the authors could emphasize. Researchers from various disciplines may use different platforms such as GitHub, Zenodo, or protocols.io, for instance, which were not monitored.

 Expanding the scope of behavioral metrics in future studies would provide a more accurate and comprehensive picture of the intervention’s impact. Additionally, qualitative follow-ups like interviews or focus groups could shed light on the specific barriers students face when attempting to implement OS practices, offering a richer contextual understanding of why behavior change may lag behind attitudinal shifts.

 Another limitation lies in the use of a non-validated questionnaire to assess perceptions and knowledge. Although the instrument was piloted, it lacks formal validation, which may affect the reliability and interpretability of the findings. A validated survey tool would strengthen future studies and ensure consistency across different institutional or cultural contexts. This is particularly important given the study’s goal of influencing policy and educational programming. By collaborating with psychometric experts to refine and validate the questionnaire, future research can generate more robust and generalizable data.

 Moreover, the intervention was delivered at a single institution with a diverse yet localized cohort, limiting the generalizability of the results. Student engagement may also have been influenced by self-selection bias, as those with a prior interest in research integrity may have been more likely to participate. Future efforts could expand the program to multiple institutions and adapt it to different disciplinary norms to enhance external validity. Tailoring the content more specifically to the research practices and challenges of individual fields might also improve relevance and uptake.

 Despite these limitations, the course successfully prompted participants to re-evaluate the value of traditional versus non-traditional research practices and the adequacy of current research evaluation systems. This shift in perception is a critical first step toward cultural change, but perception alone is insufficient to effect behavioral transformation. To bridge this gap, future interventions should not only educate but also integrate OS practices into institutional norms and researcher evaluations.

Is the work clearly and accurately presented and does it cite the current literature?

Yes

If applicable, is the statistical analysis and its interpretation appropriate?

Yes

Are all the source data underlying the results available to ensure full reproducibility?

Yes

Is the study design appropriate and is the work technically sound?

Partly

Are the conclusions drawn adequately supported by the results?

Yes

Are sufficient details of methods and analysis provided to allow replication by others?

Yes

Reviewer Expertise:

Metascience, open science/science reform, research methodology.

I confirm that I have read this submission and believe that I have an appropriate level of expertise to confirm that it is of an acceptable scientific standard, however I have significant reservations, as outlined above.

# Response

**Published:** 2025-10-16

**Authors:** Jaisson Cenci

**Affiliations:** Dentistry, Radboudumc Department of Dentistry, Nijmegen, Gelderland, The Netherlands

**Dear Dr. Sarahanne M. Field** **,**

 Thank you very much for your thoughtful and encouraging review. We are grateful for your recognition of the relevance of our work and the constructive suggestions you provided. In response to your comments, we have made several additions and clarifications in the manuscript:

- We reinforced in the discussion that the absence of a control group limits our ability to attribute attitudinal changes exclusively to the intervention.
- We revised our interpretation of OSF account creation, framing it as an introductory step rather than a true behavioural shift.
- We added a remark in the limitations section emphasizing that relying solely on OSF may have underestimated the extent of open science engagement across disciplines. We made it clear that future research should track behavioural changes through a broader set of platforms and metrics.
- We now highlight the value of follow-up with qualitative methods, such as interviews or focus group discussions, to better understand barriers to behavioural change.
- We acknowledged that the questionnaire, although piloted, was not formally validated, and we recommend future validation with psychometric expertise.
- We addressed the limitation of self-selection bias and the context-specific nature of the cohort, suggesting the need for broader, multi-institutional studies.
- Finally, we now emphasize in the conclusion that educational efforts alone are insufficient for sustained behavioural change, and must be paired with institutional and structural incentives.

Thank you again for your careful review and for helping us improve the manuscript.

# Reviewer report: Reviewer response for version 1

**DOI:** 10.5256/f1000research.171052.r375970

**Published:** 2025-05-08

**Authors:** Joachim Schöpfel

**Affiliations:** 1 University of Lille, Villeneuve-d’Ascq, France

The article reports the findings of a single-group pre-post study designed to evaluate the impact of an online training program on improving adherence to, knowledge of, and perceptions regarding the adoption of open science practices and responsible research practices among graduate researchers at the Brazilian Universidade Federal de Pelotas. According to the results, while the program had minimal to no impact on participants' actual implementation of open science and responsible research practices, it did lead to an improvement in their perceptions of open science and their stated intentions to adopt such practices in the future.

 The training program and associated research project are described appropriately. However, the review of the existing literature on the education of graduate and PhD students in open science, research ethics, and integrity is based on a rather limited selection of sources. This narrow literature base should be expanded to better contextualize the study and enrich the discussion of its findings.

 From the outset, the paper should clearly define its target population. Throughout the text, there is a lack of consistency in the use of terminology, with the study shifting between referring to early career researchers, PhD students, and Master’s students. For instance, Figure 1 references only PhD students, whereas the final sample includes over 50% Master’s students—who, in many academic contexts, are not classified as early career researchers and often have limited or no research experience. While this does not imply that Master’s students do not require training in open science, research integrity, and ethics, their academic environment, practical engagement in research, and the potential outcomes of such training can differ significantly from those of PhD students. Assessing both groups together likely diminishes the overall clarity and strength of the study's findings.

 The design of the study is generally suitable for research in educational settings. Statistical significance is appropriately calculated. Nonetheless, there are at least three notable limitations that undermine the academic rigor of the article. First is the absence of a control group. Second is the brief interval between the pre- and post-assessment phases. Third, the sample itself may not be ideal for evaluating changes in research practices due to its heterogeneity and the early career stage of participants, many of whom have limited opportunities or incentives to engage in research. The authors acknowledge these limitations in their discussion.

 The project and its data analysis are documented with sufficient transparency to allow replication by other researchers. While the modules, key content, and activities of the online training are summarized in a table with limited detail, a more comprehensive course outline is openly accessible via the OSF platform.

 Although the statistical methods employed are appropriate for the study’s scope, the design limitations—including the absence of a control group, the short follow-up period, and the sample’s diversity—preclude a causal interpretation of the (partial lack of) effects observed in the training intervention.

 The source data supporting the study’s findings are available through the OSF platform, ensuring transparency and facilitating future research.

 As previously noted, the authors demonstrate an awareness of their study’s limitations and are accordingly cautious in their conclusions. However, the discussion section would benefit from being more clearly structured, ideally divided into three or four subsections. Additionally, a separate conclusion section should be added, providing a concise summary of the main findings, key takeaways, and recommendations for future research and training initiatives.

Is the work clearly and accurately presented and does it cite the current literature?

Partly

If applicable, is the statistical analysis and its interpretation appropriate?

Yes

Are all the source data underlying the results available to ensure full reproducibility?

Yes

Is the study design appropriate and is the work technically sound?

Partly

Are the conclusions drawn adequately supported by the results?

Yes

Are sufficient details of methods and analysis provided to allow replication by others?

Yes

Reviewer Expertise:

information sciences

I confirm that I have read this submission and believe that I have an appropriate level of expertise to confirm that it is of an acceptable scientific standard, however I have significant reservations, as outlined above.

# Response

**Published:** 2025-10-16

**Authors:** Jaisson Cenci

**Affiliations:** Dentistry, Radboudumc Department of Dentistry, Nijmegen, Gelderland, The Netherlands

**Dear** **Dr. Joachim Schöpfel,**

 Thank you for your constructive and insightful feedback. We truly appreciate your time and careful evaluation of our manuscript. Below, we address each of your points in detail:

- We acknowledge the limitations regarding the literature base and have revised the introduction and discussion to include a broader set of studies on educational interventions related to open science, research ethics, and research integrity in graduate education. This includes additional references covering training programs, challenges, and best practices in various disciplines and countries.
- Thank you for highlighting this important inconsistency in terminology. We have revised the manuscript to clearly define our target population as graduate students (both Master’s and PhD), and we now consistently refer to them as such throughout the paper. We also corrected Figure 1 for terminology. Additionally, we have added an explanation to the Discussion, more specifically to the limitation section, addressing how the inclusion of Master’s students may have influenced the findings, considering their earlier research stage and lower engagement in formal research projects.
- We have expanded the limitations section to reflect the concerns you mention more explicitly: the absence of a control group, the short follow-up period, and the heterogeneity of the participants.
- Based on your valuable suggestion, we restructured the Discussion section into clear subsections to improve readability and clarity. We also included a new “Conclusion” section summarizing the key findings, limitations, and implications for future research and training programs.

We are grateful for your recommendations, which we believe have substantially improved the clarity and rigor of our manuscript.

# Reviewer report: Reviewer response for version 1

**DOI:** 10.5256/f1000research.171052.r373115

**Published:** 2025-04-24

**Authors:** Juan-José Boté-Vericad

**Affiliations:** 1 Universitat de Barcelona, Barcelona, Catalonia, Spain

Authors present a pre-post intervention study on the impact of an educational course promoting open science (OS) and responsible research practices (RRPs) among graduate students at a Brazilian university. The course included approximately 85 hours of lectures and activities, both asynchronous and synchronous, and was designed to encourage graduate students to understand and adopt practices such as study registration, protocol sharing, and data transparency.

 A total of 80 students participated. The study collected data on both real behavior (measured through the number of Open Science Framework (OSF) accounts and outputs) and self-reported perceptions and intentions (using a detailed questionnaire). The results showed that while students' creation of OSF accounts increased dramatically, there was very little change in other behaviors (such as dataset sharing or preprint posting). However, there were positive changes in how students viewed OS and RRPs and in their intentions to use these practices in the future.

 The article is well organized and clearly written. It includes all the necessary background, defines the main terms used (like OS and RRPs), and explains why the topic is important. Authors cite key and recent publications, including those from DORA, the Hong Kong Principles, and UNESCO, which shows they are aware of the ongoing discussions in the field. The course itself was well designed, with clearly defined modules and practical activities. Including synchronous workshops and real engagement with the OSF platform adds value to the intervention. The conclusions are careful and balanced. The authors clearly state that the course had a stronger effect on changing perceptions and future intentions than on actual behavior.

 The questionnaire was piloted but not formally validated, which the authors mention as a limitation. To increase confidence in the findings, a full psychometric validation (e.g., internal consistency, factor analysis) would be recommended in future studies. Also, to better understand why students did not adopt certain practices (like data sharing or preprints), a small number of follow-up interviews could have provided useful context.

 Some small amendments

 While the abstract mentions that OSF outputs "showed little or no improvement", it might be helpful to clearly state that no new registrations, datasets, or preprints were added within 12 months.  Mention and explain the questionnaire limitations earlier. The article acknowledges that the questionnaire is not validated, but this point is buried in the discussion.

 Provide more detail on the institutional context adding a short paragraph on the broader institutional support (or lack thereof) for OS practices at UFPel.

 Add a short table summarizing the questionnaire structure. Since the text explains the five parts of the questionnaire, a simple table would help readers quickly see the structure.

Is the work clearly and accurately presented and does it cite the current literature?

Yes

If applicable, is the statistical analysis and its interpretation appropriate?

Yes

Are all the source data underlying the results available to ensure full reproducibility?

Yes

Is the study design appropriate and is the work technically sound?

Yes

Are the conclusions drawn adequately supported by the results?

Yes

Are sufficient details of methods and analysis provided to allow replication by others?

Yes

Reviewer Expertise:

My research are in Open Science in the Librarianship and Information Science field.

I confirm that I have read this submission and believe that I have an appropriate level of expertise to confirm that it is of an acceptable scientific standard.

# Response

**Published:** 2025-10-16

**Authors:** Jaisson Cenci

**Affiliations:** Dentistry, Radboudumc Department of Dentistry, Nijmegen, Gelderland, The Netherlands

**Dear Dr. Juan-José Boté-Vericad,**

 Thank you for your generous and supportive comments on our manuscript. We appreciate your recognition of the relevance and clarity of the work, and your suggestions for further improving the manuscript’s structure and transparency. In response to your points:

- We have revised the abstract to clarify that, although OSF account creation increased substantially, no new registrations, datasets, or preprints were added within the 12-month follow-up period.
- We have reinforced the lack of formal validation of the questionnaire as you suggested.
- About the table summarizing the five sections of the questionnaire you mentioned, we felt that this would make the manuscript too long. Still, we inserted a direct link to the entire questionnaire available at OSF.

Thank you again for your insightful feedback and kind words, which helped us further improve the clarity and completeness of the manuscript.
